# Purification and Partial Characterization of a New Antitumor Protein from *Tegillarca granosa*

**DOI:** 10.3390/md13031466

**Published:** 2015-03-17

**Authors:** Shuangshuang Lv, Jingjing Gao, Ting Liu, Jianhua Zhu, Jian Xu, Liyan Song, Jincai Liang, Rongmin Yu

**Affiliations:** 1 Biotechnological Institute of Chinese Materia Medica, Jinan University, Guangzhou 510632, China; E-Mails: 13691860569@163.com (S.L.); gjj0509@126.com (J.G.); lenovo1688@126.com (J.X.); yffsljc2006@sina.com (J.L.); 2 Department of Natural Medicinal Chemistry, Jinan University, Guangzhou 510632, China; E-Mail: liutina@sina.cn; 3 Department of Pharmacology, Jinan University, Guangzhou 510632, China

**Keywords:** *Tegillarca granosa*, protein, purification, characterization, antitumor activity

## Abstract

A new protein, coded as D2-3, was obtained from the marine organism *Tegillarca granosa L.* by anion exchange and hydrophobic chromatography. The purity of D2-3 was over 99.0% as measured by RP-HPLC. Its molecular weight was shown to be 20.320 kDa by ESI-MS/MS, and the isoelectric point of D2-3 was 4.70. The antitumor activity of D2-3 against four human tumor cell lines was measured by MTT assay. The conformational structure of D2-3 was further characterized by UV-vis, FT-IR and CD spectroscopy. Partial amino acid sequences of D2-3 were determined to be LMMTDVEESR, SSHMLSECRRK, KNGRNVDISHKDKG, SSDPTLMDPDDTNKDR, SSDKNTCSKTEYYTR and SSETMPYDVLDTNEMR via MALDI-TOF-MS and *de novo* sequencing.

## 1. Introduction

Covering about 70% of the earth’s surface, the ocean is known to be the cradle of life and harbors approximately 80% of all living organisms on earth. Given the special marine environment, various products with therapeutic application have been generated from marine organisms [1]. Up to now, several bioactive peptides and depsipeptides have been studied in depth, and even have been taken to clinical study levels [2], such as Didemnin B [2], Aplidine [3], Geodiamolide H [4] and kahalalide F [5], which have already entered clinical trials.

Among marine animals, mollusks are the most important source of active substances. Mollusks, with more than 130,000 species, are the second largest group of animals containing high levels of protein. Experience has shown mollusks to be a rich reservoir of structurally diverse bioactive compounds which possess antitumor, antihypertensive, antioxidant and immunomodulatory activities. About ten percent of these products have antitumor activities, and research has focused on macromolecule constituents such as proteins, polypeptides and polysaccharides [6]. Various substances extracted from mollusks such as mercenene, paolin, and an alone polysaccharide, were reported to exhibit significant antitumor activities [7]. Anti-bacterial peptides including defensin and mytilin were also found to possess the ability to inhibit tumor growth [8]. A protein with the molecular weight of 76 kDa which was extracted from *Meretrix meretrix* could be active against L1210 lymphocytic leukemia [9]. Therefore, shellfish proteins were regarded as a rich potential resource for the discovery and development of potential antitumor drugs.

*Tegillarca granosa L.* (*Arcagranosa L*.), a bivalve mollusk from the Arcidae family, is widely distributed on China’s east coast and in Southeast Asia [10]. It is a major fishery and aquaculture species with proteins, various vitamins and amino acids, resulting in its use in traditional Chinese medicine and seafood [11]. *T. granosa* has been well researched in the field of genetics, which includes cDNA cloning and gene expression, as well as the functional study on the immune expression of hemoglobin gene [12]. In recent years, it has been reported that *T. granosa* extracts contained several active protein components [13], with diverse activities against fatigue [14], coagulation [15] and tumor growth [16,17]. Despite these findings, the purified protein with antitumor activity from *T. granosa* has not been reported.

The goal of this investigation is to purify the antitumor protein from *T. granosa*, to determine its physicochemical and structural properties, and to evaluate its inhibitory effect on the proliferation of human tumor cell lines.

## 2. Results and Discussion

### 2.1. Separation of Antiproliferative Protein

In this study, we extracted and purified a new protein with significant antitumor activity from *T. granosa*. To obtain the natural proteins, all the experiment procedures were controlled at 4 °C. The total proteins were fractionated by salting-out at increasing saturation levels of ammonium sulfate. Fraction L1, fraction V2, fraction V3 and fraction J3-1 were obtained at various ammonium sulfate saturations. The yield of the crude protein (J3-1) extracted at 70%–100% saturation of ammonium sulfate was 0.26%, based on weight of wet visceral mass.

In order to purify the active protein components from *T. granosa*, consecutive chromatographic methods were applied along with the detection of the antitumor activities by 3-(4,5-dimethylthiazol-2-yl)-2,5-diphenyl-etrazolium bromide (MTT) assay. As shown in Table 1, the crude protein, J3-1, suppressed the proliferation of HT-29, HepG2, HeLa and A549 cells with the IC_50_ values less than 500 μg/mL. Furthermore, the content of protein and saccharide in J3-1 was determined to be 59.55% and 5.76%, respectively. Therefore, J3-1 was chosen for further separation and purification.

**Table 1:** Antiproliferative activities of protein samples against four tumor cell lines (IC_50_, μg/mL ± SD, *n* = 3).

	IC_50_ (µg/mL)			
	HT-29	HepG2	HeLa	A549
L1	418.2 ± 122.1	>500	>500	>500
V2	490.9 ± 161.0	>500	>500	>500
V3	461.7 ± 212.4	>500	>500	>500
J3-1	346.2 ± 43.0	497.2 ± 74.3	494.2 ± 81.9	407.7 ± 63.5
D1	>500	>500	>500	>500
D2	154.8 ± 12.6	422.1 ± 32.7	480.6 ± 22.5	366.8 ± 41.7
D3	222.9 ± 42.2	>500	>500	>500
D2-1	>500	>500	>500	>500
D2-2	83.7 ± 19.6	>500	>500	>500
D2-3	25.4 ± 1.2	281.0 ± 19.8	271.3 ± 15.1	235.2 ± 20.5
Cisplatin	7.59 ± 0.09	3.22 ± 0.02	1.56 ± 0.08	0.79 ± 0.05

Fraction L1: The supernatant after homogenate; Fraction V2: The precipitate after 0%–35%; (NH_4_)_2_SO_4_ saturation; Fraction V3: The precipitate after 35%–70% (NH_4_)_2_SO_4_ saturation; Fraction J3-1: The precipitate after 70%–100% (NH_4_)_2_SO_4_ saturation.

**Figure 1:**
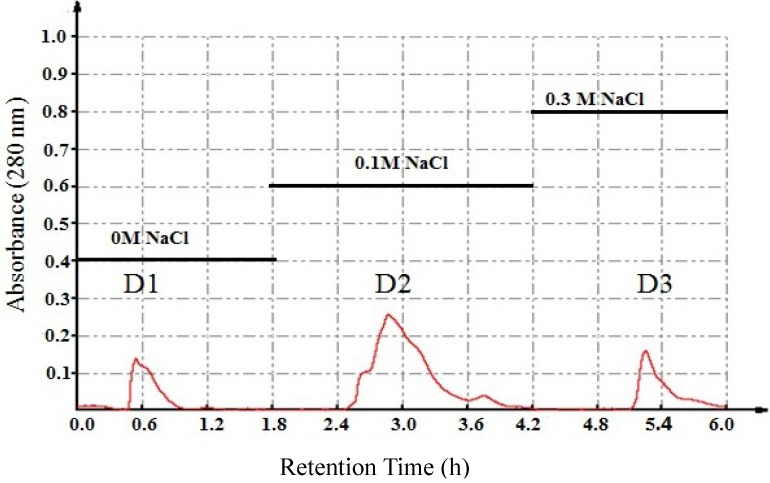
Purification of J3-1 by DEAE Sepharose Fast Flow anionexchange chromatography.

Three peaks named D1, D2 and D3 (Figure 1) were obtained from DEAE Sepharose Fast Flow column. In the evaluation of *in vitro* antitumor activity for the three fractions, fraction D2 was revealed to have significant inhibitory activity on the proliferation of tumor cells, especially the proliferation of HT-29 cells (IC_50_ = 154.8 μg/mL). The other two fractions showed weaker inhibitory activity on tumor cells’ proliferation. Furthermore, D2 was shown to possess the highest protein content at over 78%, while D1 and D3 had protein contents of less than 70%. This result proposed that D2 was the active component best suited for further separation.

**Figure 2:**
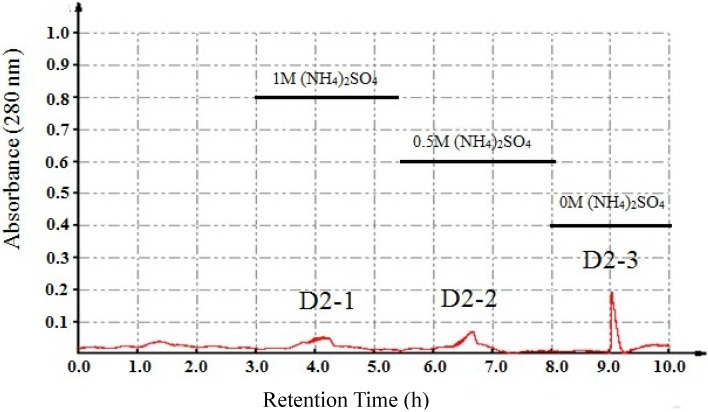
Purification of D2 by Phenyl Sepharose CL-4B hydrophobic chromatography.

As shown in Figure 2, D2 was loaded in Phenyl Sepharose CL-4B hydrophobic chromatography column, and three peaks (D2-1, D2-2 and D2-3) were collected. The results of the antiproliferation assay showed that D2-3 had significant inhibitory effects among all the fractions, with the IC_50_ values of 25.4, 281.0, 271.3 and 235.2 μg/mL against HT-29, HepG2, HeLa and A549 cell lines, respectively. D2-3 exhibited the strongest antitumor activity, with an IC_50_ value against HT-29 cells that was about 13-fold lower than that of the crude protein (J3-1).Therefore, further investigation of D2-3 was warranted.

### 2.2. Characterization of Purified Protein

**Figure 3:**
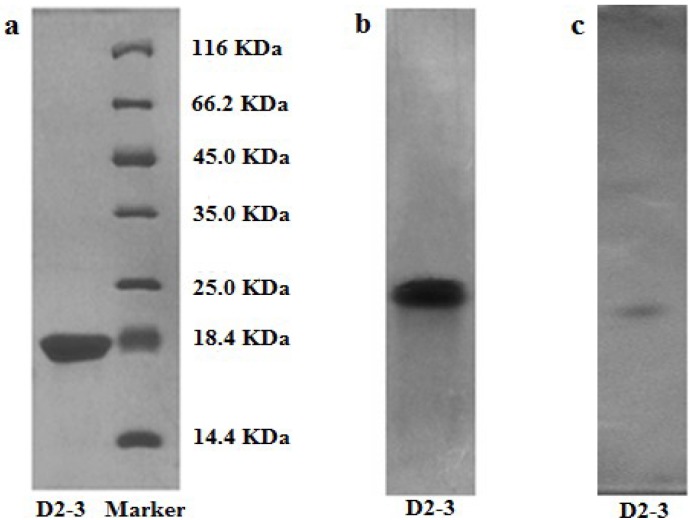
Electrophoretograms of D2-3. (**a**) SDS-PAGE profile of D2-3; (**b**) Native-PAGE profile of D2-3; (**c**) IEF-PAGE profile of D2-3.

**Figure 4:**
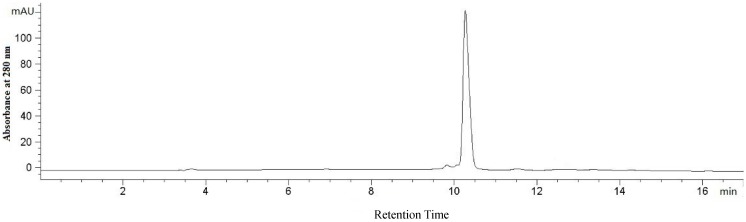
RP-HPLC profile of D2-3.

The protein obtained from *T. granosa* was analyzed by polyacrylamide gel electrophoresis to determinate its molecular weight and purity. A single band of purified protein under denaturing and non-denaturing conditions was detected by sodium dodecyl sulfate polyacrylamide gel electrophoresis (SDS-PAGE) and native polyacrylamide gel electrophoresis (Native-PAGE), respectively. The results showed that D2-3 was electrophoretically homogeneous, giving a single band in SDS-PAGE and Native-PAGE (Figure 3a,b). In addition, SDS-PAGE analysis revealed that the molecular weight of D2-3 was approximately 20 kDa, with the molecular weight markers ranging from 14.4 to 116 kDa as standard. Similarly, a single sharp peak of D2-3 was located on RP-HPLC elution profile (Figure 4), and the purity of D2-3 was over 99.0%. The information also demonstrated that D2-3 had been purified to homogeneity. According to isoelectric point calibration curves, the result of isoelectric focusing-polyacrylamide gel electrophoresis (IEF-PAGE) showed that D2-3 was a single band and isoelectric point was 4.70 (Figure 3c).

**Figure 5:**
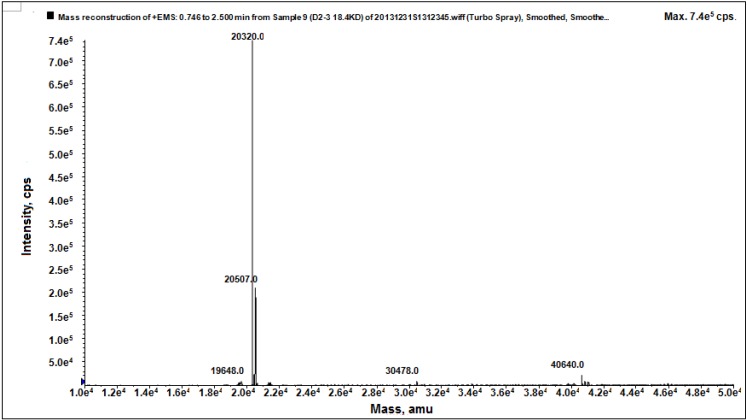
Mass spectrum of D2-3.

As shown in Figure 5, the precise molecular weight of D2-3 was determined to be 20.320 kDa by electrospray ionization mass spectrometry (ESI-MS/MS), which was consistent with the result of SDS-PAGE.

**Figure 6:**
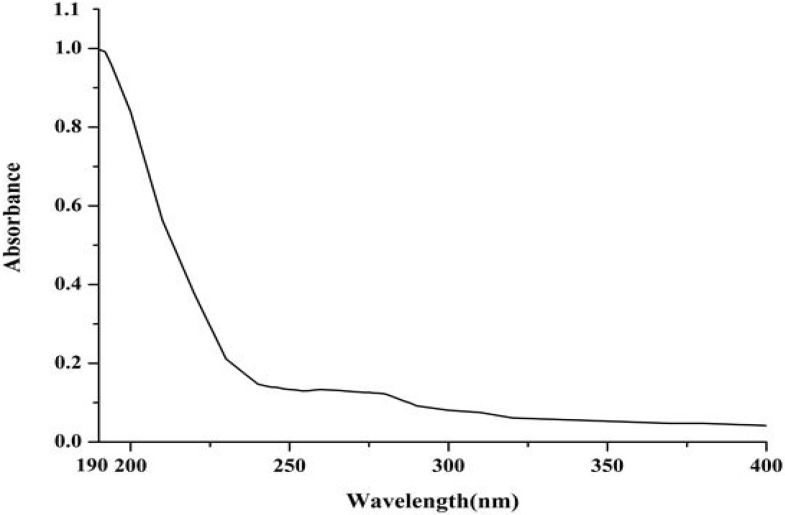
UV spectrum of D2-3.

**Figure 7:**
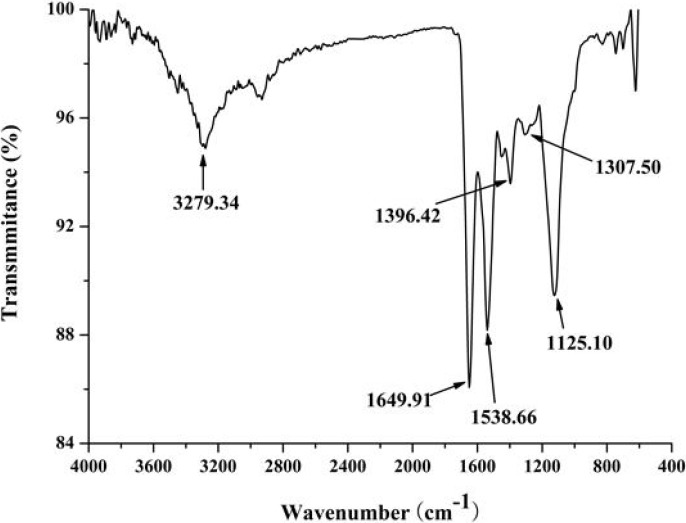
FT**-**IR spectrum of D2-3.

**Figure 8:**
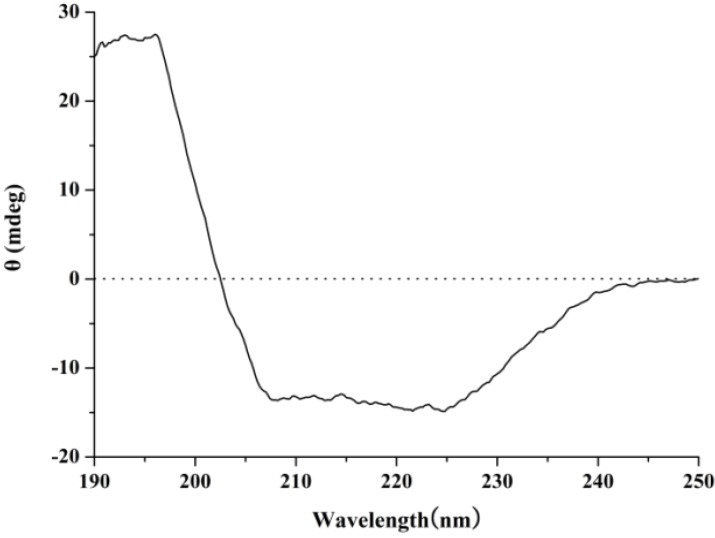
Circular dichroism spectrum of D2-3.

The ultraviolet-visible (UV-vis) absorption, Fourier transform infrared (FT-IR) and circular dichroism (CD) spectroscopies were applied to investigate the structure features of purified protein. The UV-vis absorption spectrum of a 0.1 mg/mL solution of D2-3 in distilled water was determined. D2-3 performed special absorption at 190 nm and 275 nm (Figure 6), which was the typical absorption of peptide bond and amino acid residues such as tyrosine and tryptophan, respectively [18]. FT-IR spectroscopy has been recognized as a valuable and sensitive tool for the examination of protein conformation [19,20]. The amide I band is the sum of all the contributions caused by the secondary of the protein (α-helix, β-sheets, turns and unordered structures) [21]. The FT-IR spectrum of D2-3 is shown in Figure 7. The result indicated that the intense absorption frequencies characterized at 1649.91, 1538.66 and 1307.50 cm^−1^ were amide I, II and III, respectively. The strong absorption band observed at about 1650 cm^−1^ was assigned to α-helix [21]. CD spectroscopy provides rapid determinations of protein secondary structure. The CD spectrum (Figure 8) of D2-3 showed two weak negative bands at 208 and 225 nm, and an intense wave at 196 nm. Then the secondary structure of D2-3 was analyzed and calculated using the Jasco protein secondary structure estimation program, and the results showed that it contained 58.2% α-helix, 20.1% β-turn and 21.7% random coil. The total content of α-helix and β-turn accounted for over 70% in the secondary structure. Hence, we regarded D2-3 as a highly ordered and stable protein.

Sequence information for D2-3 was acquired via a matrix-assisted laser desorption/ionization time-of-flight mass spectrometry (MALDI-TOF-MS) instrument. Based on the manual calculation of the molecular weights and the *m/z* values, some information about the amino acid sequences of D2-3 was obtained: the sequence of fragment ion *m/z* 1210.57 was LMMTDVEESR, fragment ion *m/z* 1406.70 was SSHMLSECRRK, fragment ion *m/z* 1567.76 was KNGRNVDISHKDKG, fragment ion *m/z* 1822.90 was SSDPTLMDPDDTNKDR, fragment ion *m/z* 1839.92 was SSDKNTCSKTEYYTR, and fragment ion *m/z* 1870.89 was SSETMPYDVLDTNEMR (see Figure S1 of Supplementary Information). Peptide partial sequences of D2-3 were gathered by integrating the six fragments and performed for further analysis.

**Figure 9:**
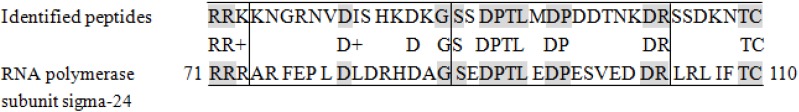
The 71 to 110 amino acid sequence alignments of RNA polymerase subunitsigma-24.

Based on the analysis by the BLAST search program, the comparison of amino acid sequences between the purified protein (D2-3) and a known protein (RNA polymerase subunit sigma-24) is presented in Figure 9. The values of total score, positives and identities were 35.0, 50% and 40%, respectively (Table 2). The alignment of the amino acid sequence of D2-3 had only 4.8% homology with that of RNA polymerase subunit sigma-24. This suggests that D2-3 is a newly discovered protein that has not been extensively characterized previously.

**Table 2:** Peptide sequences identified by BLAST software.

Protein Description	Sequence ID	Score	Expect	Identities	Positives	Gaps
RNA polymerase subunit sigma-24	WP 029888572.1	35.0	4.3	16/40 (40%)	20/40 (50%)	0/40

No effective signal peaks were available in the determination of N-terminal sequence (see Figure S2 of Supplementary Information). Five amino acid residues (Gly-Pro-Tyr-Tyr-Tyr) were analyzed, but their amount yield (pmol) and evaluated values were very low. Therefore, we suppose that the *N*-terminus of the purified protein (D2-3) was blocked.

## 3. Experimental Section

### 3.1. Sample and Materials

Fresh *T. granosa* was collected from Huangsha seafood market in Guangzhou (China), and authenticated by Rongmin Yu (Jinan University, Guangzhou, China). The visceral mass of *T. granosa* was separated and stored at −20 °C.

DEAE Sepharose Fast Flow and Phenyl Sepharose CL-4B were purchased from GE Healthcare. Sodium dodecyl sulfate (SDS), trishydroxymethylaminomethane (Tris), l-glutamide and MTT were obtained from Sigma Chemical Co. (St. Louis, MO, USA). RPMI-1640 medium was purchased from Hyclone(Logan, UT, USA) and Cisplatin (cis-diamminedichloro-platinum, CDDP) from Sigma (St. Louis, MO, USA). Acetonitrile was of chromatographic grade. Other reagents were all of analytical grade.

### 3.2. Extraction of Crude Protein

The visceral mass of *T. granosa* (200 g) was washed with distilled water three times, followed by homogenizing with triple volume of PBS (0.03 M, pH 8.0). After extraction with ultrasound at 4 °C for 40 min, the homogenate was centrifuged at 8000 rpm for 30 min at 4 °C. Then ammonium sulfate was slowly added to the supernatant with increasing concentrations. Three prepared with various saturated ammonium sulfate solutions. The precipitate of 70%–100% saturation was re-dissolved in the above buffer and dialyzed to remove ammonium sulfate for 36 h [22]. The crude protein was obtained by freeze-drying.

### 3.3. Purification of Protein

The crude protein was dissolved in 0.03 M Tris-HCl buffer (pH 8.0) with a proportion of 1:10 (w/v) before loading onto a DEAE Sepharose Fast Flow column, which was pre-equilibrated with the above buffer. The column was then eluted with 0, 0.1 and 0.3 M NaCl prepared in the same buffer at a flow rate of 1 mL/min. The absorbance was measured at 280 nm. The fractions eluted were collected, dialyzed and freeze-dried so their antiproliferative activities could be evaluated using the MTT method. The fraction with the optimal antitumor activity was loaded into a hydrophobic chromatography column.

The freeze-dried sample was dissolved in 1 M (NH_4_)_2_SO_4_ prepared with 0.05 M PBS (pH 8.0) and eluted on a Phenyl SepharoseCL-4B hydrophobic chromatography column which had been equilibrated with the above buffer. Then the column was stepwise eluted with 1.0, 0.5 and 0 M (NH_4_)_2_SO_4_ mixed in 0.05 M PBS buffer at a flow rate of 1 mL/min. The absorbance was measured at 280 nm. The protein peaks were concentrated and assayed for antitumor activity. The purified or unpurified proteins were gathered and used for further investigation.

### 3.4. Cell Culture and Cytotoxic Activity

Human colon adenocarcinoma cell line (HT-29), human lung adenocarcinoma cell line (A549), human liver carcinoma cell line (HepG2) and human cervix epithelioid carcinoma cell line (HeLa) were provided by Shanghai Institutes for Biological Sciences, Chinese Academy of Sciences, China. All cells were cultured in RPMI 1640 medium supplemented with 10% FBS and antibiotics (Penicillin 100 IU/mL, Streptomycin 100 μg/mL), and incubated at 37 °C in a humidified atmosphere containing 5% CO_2_.

Cytotoxic activity was analyzed by MTT assay *in vitro* [23]. Exponentially growing cancer cells were seeded into 96-well culture plates (4 × 10^3^ cells/well) and incubated at 37 °C in a humidified incubator with 5% CO_2_ for 24 h. After that, the culture medium was discarded and the cells were exposed to samples at different concentrations. Untreated cells were used as negative control. After incubation for 48 h, 20 μL of MTT solution (5 mg/mL) was added to each well. The plates were incubated for 4 h at 37 °C. Then, the supernatants were removed and the formazan crystals were dissolved with 200 μL of dimethylsulfoxide. The absorbance at 570 nm was recorded using a microplate reader. Cell growth inhibition was evaluated by comparing the absorbance of treated and untreated cells. All experiments were carried out in triplicate, and data in the form of mean ± SD are presented.

### 3.5. Determination of Protein and Saccharide Content

Protein concentration was estimated using the Bradford method [24]. Bovine serum albumin (BSA) was used as a standard protein. Saccharide concentration was measured by the phenol-sulfuric acid method [25,26,27] with a 100 μg/mL glucose solution as standard.

### 3.6. SDS-PAGE

Protein eluted through hydrophobic chromatography was analyzed by SDS-PAGE [28] in gel with an acrylamide concentration of 5% for the stacking gel and 16% for the separating gel. The separation was first manipulated with a voltage of 60 V for 0.5 h, and then 80 V for about 2.5 h. Protein bands were detected using the Coomassie blue staining method [29]. A middle molecular weight calibration kit (Thermo Scientific, Waltham, MA, USA) was used as a standard marker.

### 3.7. Native-PAGE

Native-PAGE was performed similarly to SDS-PAGE without SDS and the procedure was carried out at low temperature. Native-PAGE was carried out to analyze purified protein with 12% stacking gel and 5% separating gel. The electrophoresis condition was as following: 100 V for 20 min, then 160 V for 80 min. The gels were stained in Coomassie Brilliant Blue R-250.

### 3.8. IEF-PAGE

The isoelectric point of purified protein from *T. granosa* was determined by IEF-PAGE with the gel including ampholyte (40%, pH 3.5–10.0) and 5% acrylamide concentration in an electrophoresis apparatus (Protean II. BioRAD, Hercules, CA, USA). The experiment was carried out at 150 V for 0.5 h, then at 200 V for 2.5 h. The IEF-PAGE gel unloaded samples were washed with double-distilled water, and sliced into pieces of 0.5 cm in length from acidic terminal to basic terminal, then separately dipped into glass tubes containing 2.0 mL of 10 mM KCl for 30 min. The pH value of the liquid around each slice was measured. The gel-loaded samples were fixed with 10% TFA for 30 min, stained with Coomassie Brilliant Blue R-250 overnight, and then destained until faded from the background. Data were derived from the calibration curve of isoelectric points with the length of gel as abscissa and pH value as ordinate [30].

### 3.9. RP-HPLC

Sample was prepared for RP-HPLC by dissolving the protein in distilled water to 0.1 mg/mL. The protein (10 μL) was filtered and then loaded into an Agilent 1100 HPLC system fitted with a ZORBAX^®^ 300SB-C8, Agilent column (4.6 × 250 mm). The mobile phase was composed of 0.1% trifluoroacetic acid (0.1% TFA) in water (solvent A) and 0.1% TFA in acetonitrile (solvent B). The column was eluted with a gradient of 30%–70% solvent B for 30 min with the flow rate of 1 mL/min and UV detection at 280 nm.

### 3.10. Structure Elucidation

The structural feature of purified peptide was detected by UV, FT-IR, and CD spectroscopy.

UV-vis: The sample was dissolved in distilled water for the concentration of 0.1 mg/mL and measured by UV-2450 UV-vis absorption spectrophotometer (Shimadzu, Osaka, Japan). The scan range was 190–400 nm, and the distilled water was used as the blank control.

FT-IR: For FT-IR analysis, sample was dissolved in distilled water for the concentration of 0.1 mg/mL and the spectral information was measured on an EQUINOX55 spectrometer (Bruker Optics, Bremen, Germany).

CD Spectroscopy: The concentration of peptide was 0.01 mg/mL in distilled water, and the sample solution was filtered through a 0.22 μm membrane before CD analysis. Protein was measured using a Jasco J-810 spectropolarimeter (Japan Spectroscopic Co., Ltd., Hachioji, Tokyo, Japan) with a scan range of 250–190 nm [31]. All procedures were performed at 20 °C and each spectrum was recorded as the average of 3 scans [32].

### 3.11. Molecular Weight Determination

The accurate molecular weight of purified protein was determined by ESI-MS/MS. It was performed on an API type 4000 QTRAP mass spectrometer (Applied Biosystem, Foster City, CA, USA) using the conditions for protein analysis according to the reference [33].

### 3.12. Amino Acid Sequence Analysis

The amino acid sequence of peptide was identified with the reference [34]. To confirm part of the amino acid sequences of D2-3, the Coomassie-stained SDS-PAGE gel of purified D2-3 was excised and digested. Tryptic peptides were lyophilized and dissolved in 10 μL of a 50% acetonitrile/0.1% TFA solution. An amount of 0.4 μL of the sample was spotted onto the MALDI sample target plate, and then 0.4 μL of a saturated matrix solution of α-cyano-4-hydroxycinnamic acid prepared in 50% acetonitrile/0.1% TFA was added. Peptide mass spectra were obtained on a 4800 Proteomics Analyzer MALDI-TOF/TOF mass spectrometer (Applied Biosystems, Foster City, CA, USA) in the positive ion reflection mode. After an external calibration with a mixture of angiotensin II (Mr, 1046.54180), angiotensin I (Mr, 1296.68478), substance P (Mr, 1347.73543), bombesin (Mr, 1619.82235), ACTHclip 1–17 (Mr, 2093.0868), ACTH clip 18–39 (Mr, 2465.1990), Somatostatin 28 (Mr, 3147.4714), spectra were obtained in the mass range between 900 and 3500 Da with 500 laser shots. For each sample spot, a data dependent acquisition method was created to select the six most intense peaks for subsequent MS/MS data acquisition, excluding those from the matrix, due to trypsin autolysis or acrylamide peaks. MS/MS spectra were acquired with 1200 laser shots in the mass range from 10 Da to the mass of the parent ion using an interpretation method presented on instrument software, where the six most intense peaks were selected and MS/MS spectra were generated automatically. To ensure reliable identification, the results from both the MS and MS/MS spectra were used in the database search. Peptide identification was accepted when the score read by the Mascot search routine was higher than 90. The sequence of peptide fragments was determined by *de novo* sequencing using the Applied Biosystems software as presented by Yergey [35]. Homology searches were performed with BLAST in the UniProtKB database.

### 3.13. Statistical Analysis

All assays were conducted in triplicate, and the results were expressed as mean ± SD. GraphPad Prism 5.0 was used for statistical analysis.

## 4. Conclusions

A novel protein (D2-3) with antitumor activity was progressively purified from *T. granosa* by bioassay-guided isolation. The result of the MTT assay revealed that it exhibited antiproliferative activity against HT-29, HepG2, HeLa and A549 cells *in vitro*, especially against HT-29 cells with an IC_50_ value of 25.4 μg/mL. In addition, the physicochemical and structural properties of D2-3 were characterized by electrophoresis techniques as well as UV-vis, FT-IR and CD spectroscopies. All the results advanced our understanding of the structural and functional properties of D2-3, and demonstrated its promising potential for nutritional and therapeutic applications. Further studies are currently underway to elucidate the mechanism behind its antitumor activity.

## Supplementary Files

Supplementary File 1
