# Supplementary material for: Purification and Partial Characterization of a New Antitumor Protein from Tegillarca granosa
Source: Mar Drugs. 2015 Mar 17;13(3):1466–80. doi: 10.3390/md13031466 (PMC4377994; doi:10.3390/md13031466)
Supplement: Supplementary File 1 [file marinedrugs-13-01466-s001.pdf]

Supplementary Information

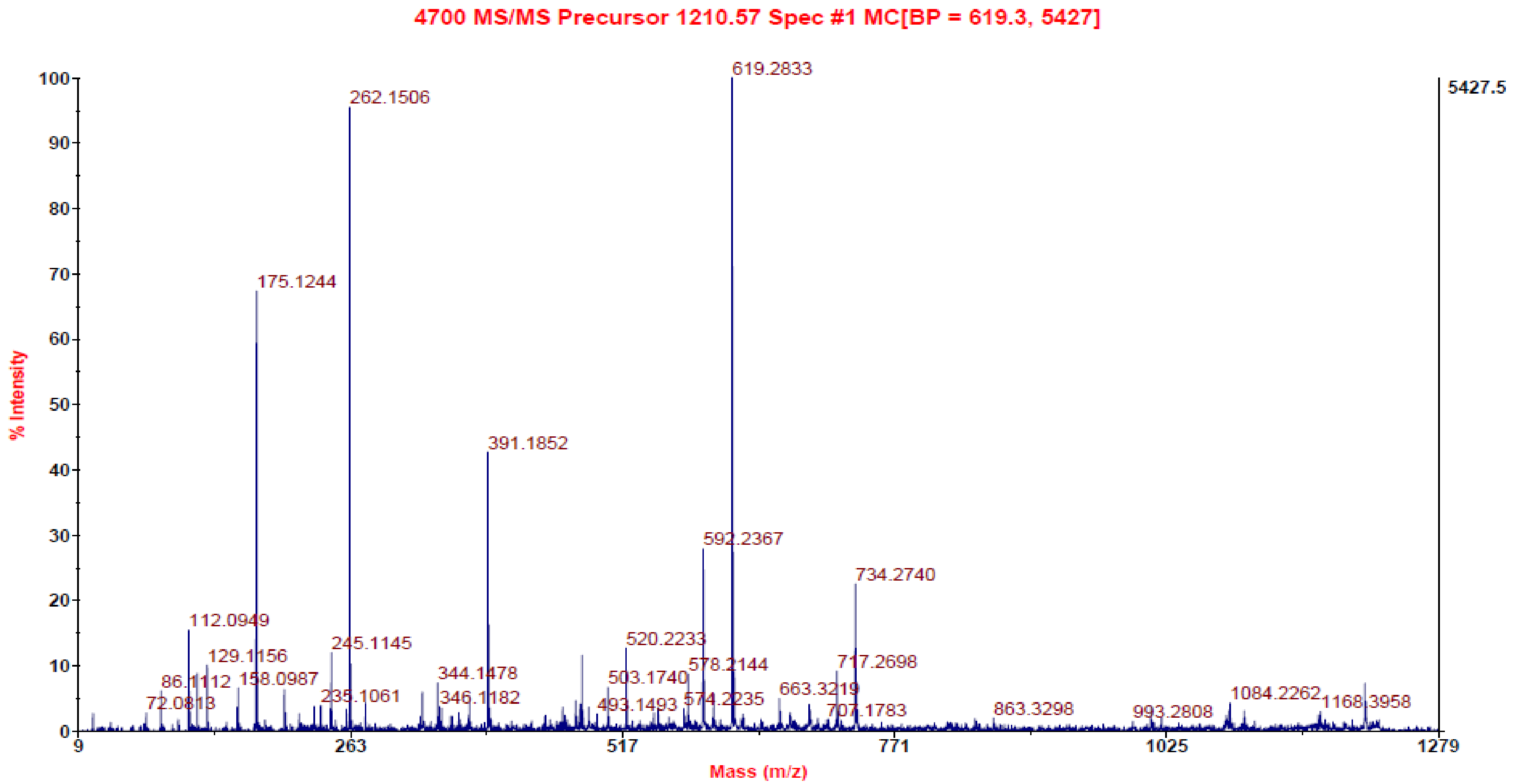

(a)

Figure S1. *Cont.*

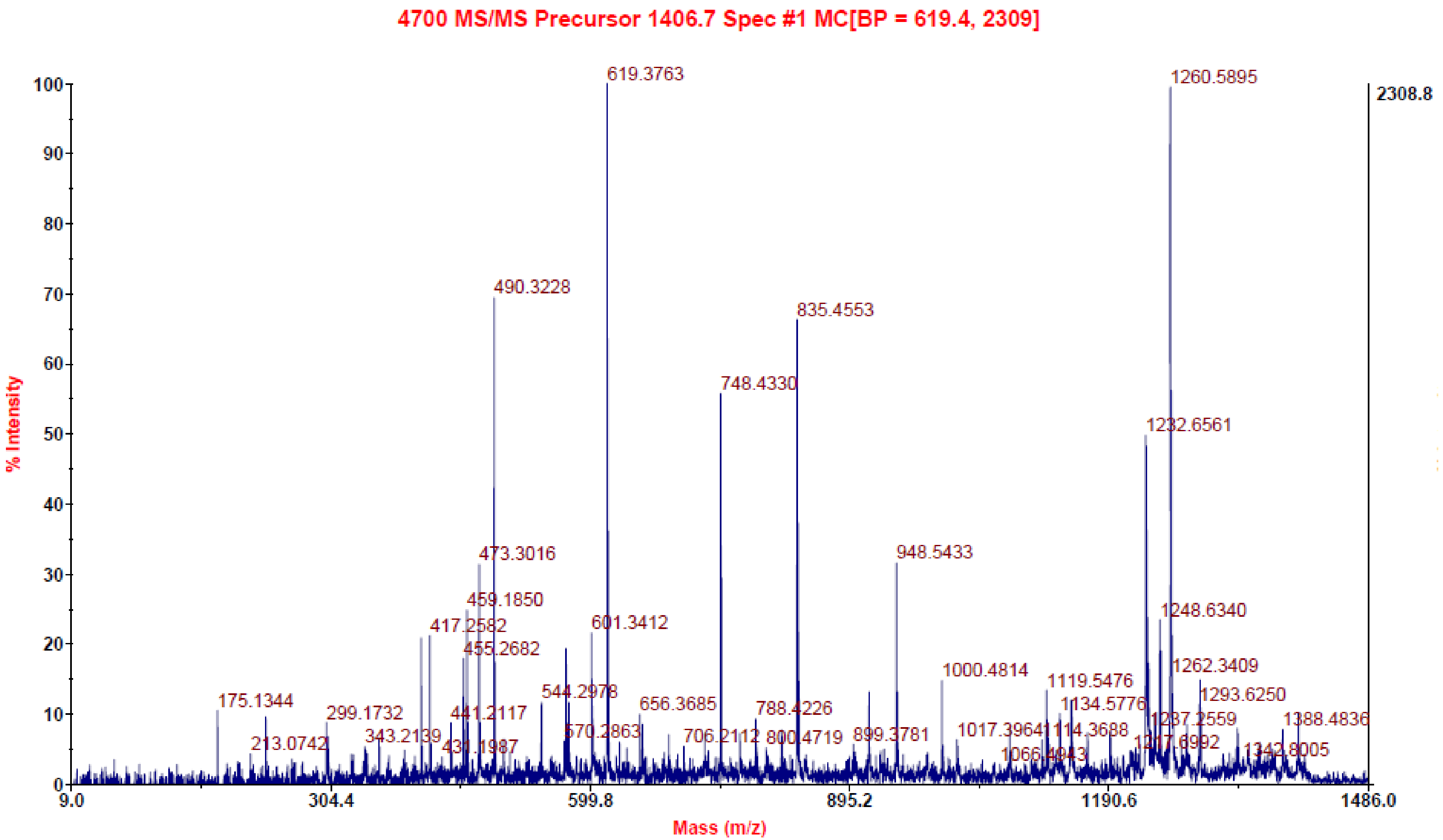

(b)

Figure S1. *Cont.*

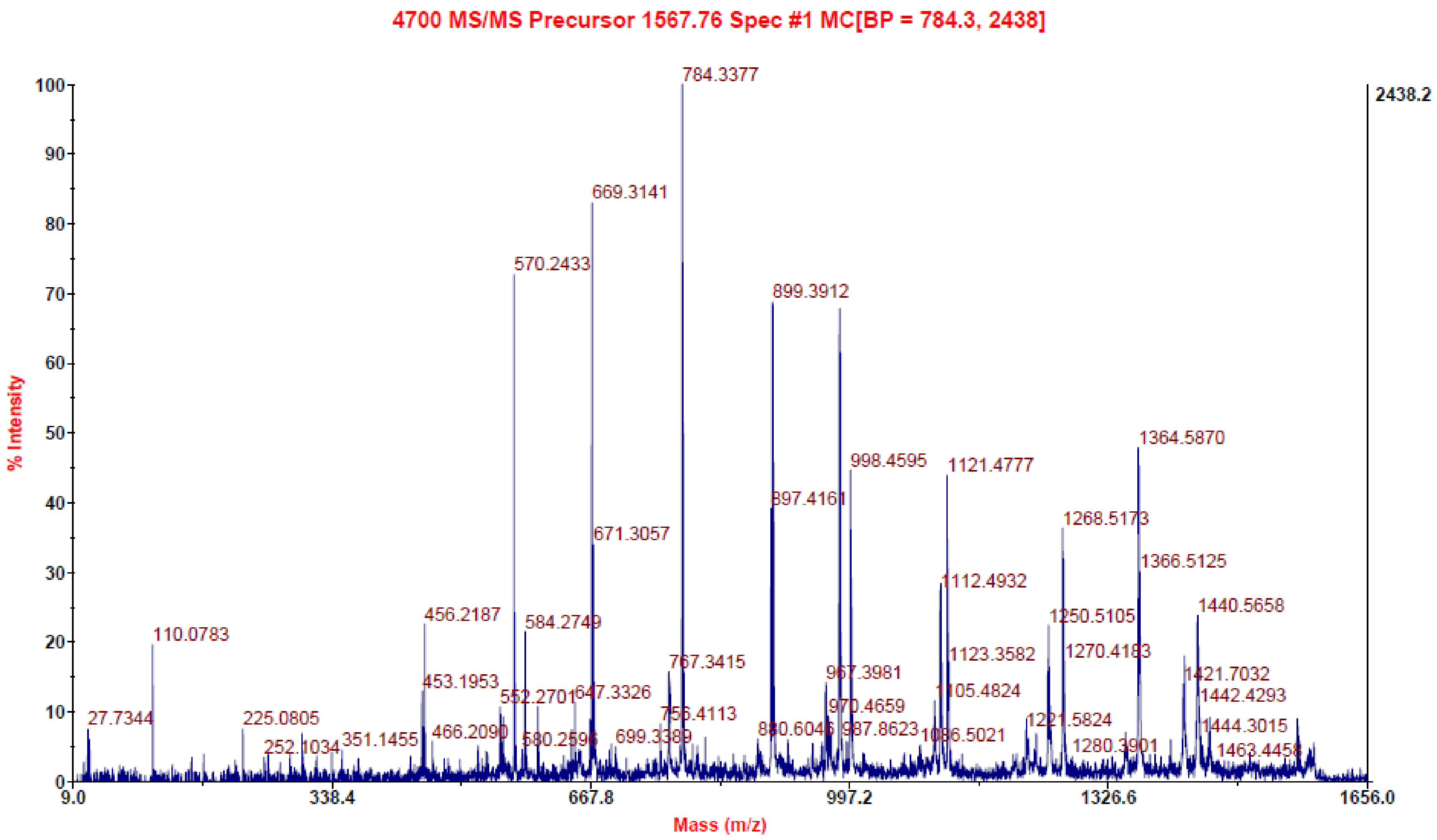

(c)

Figure S1. *Cont.*

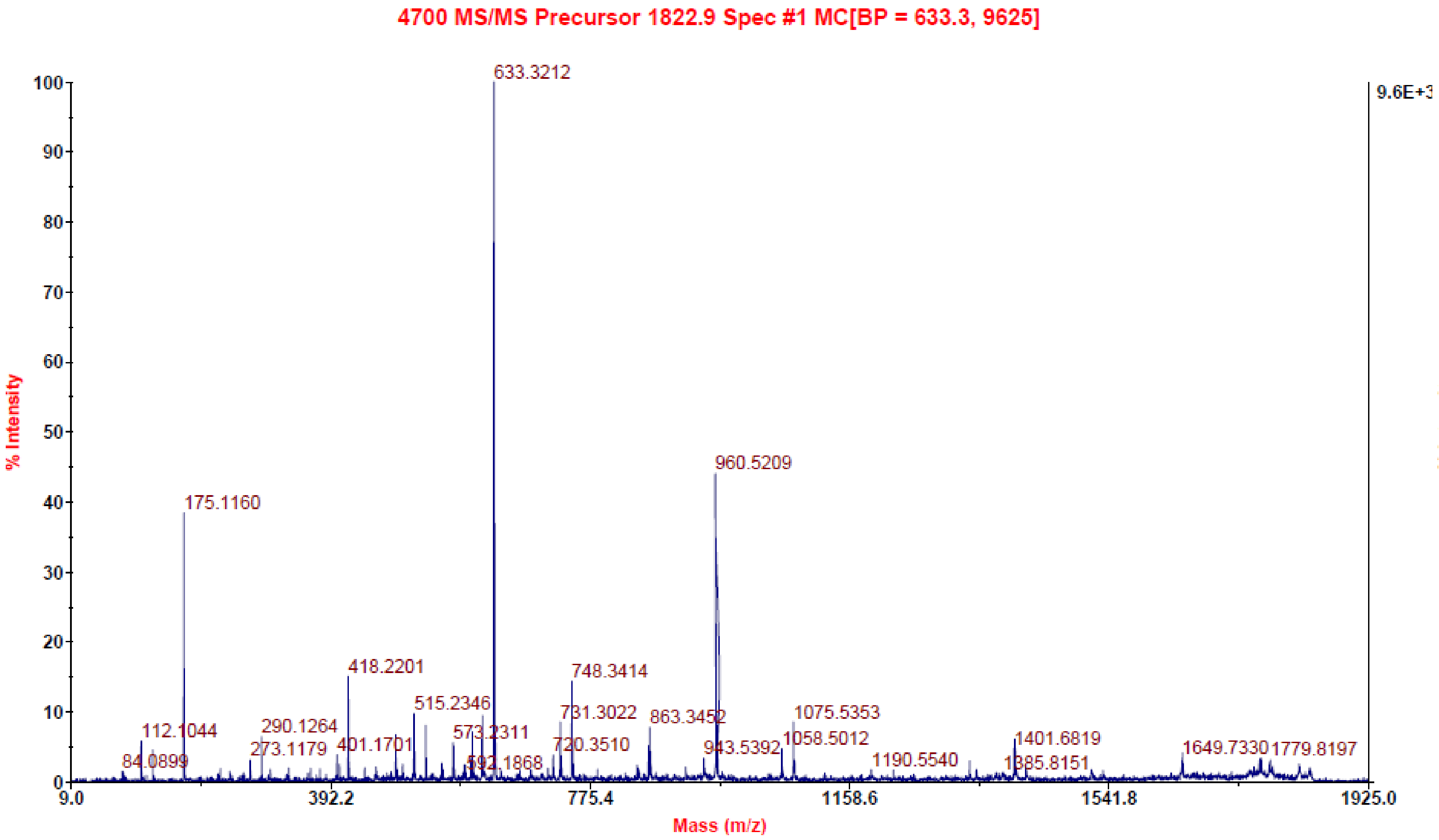

(d)

Figure S1. *Cont*.

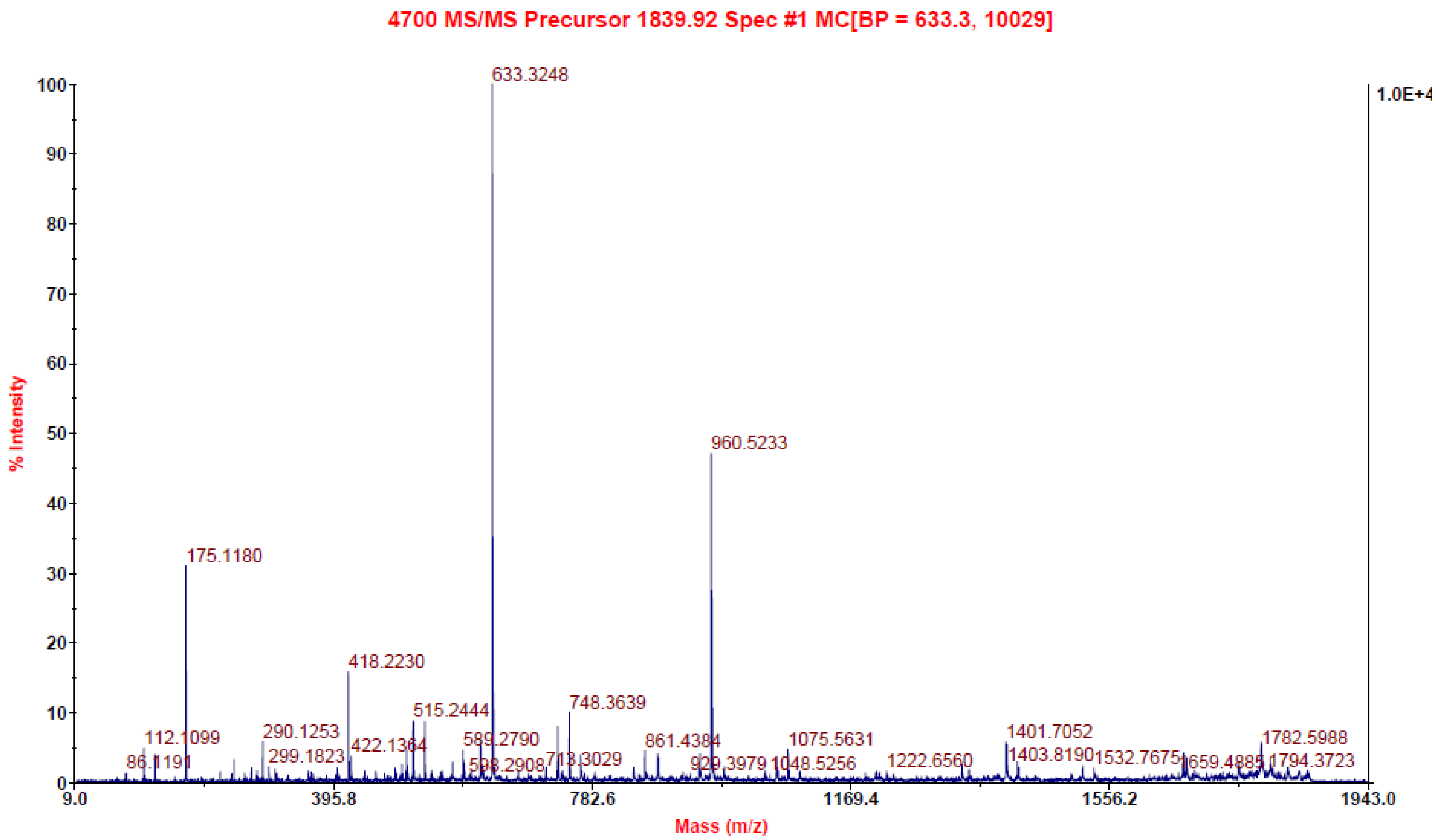

(**e**)

Figure S1. *Cont.*

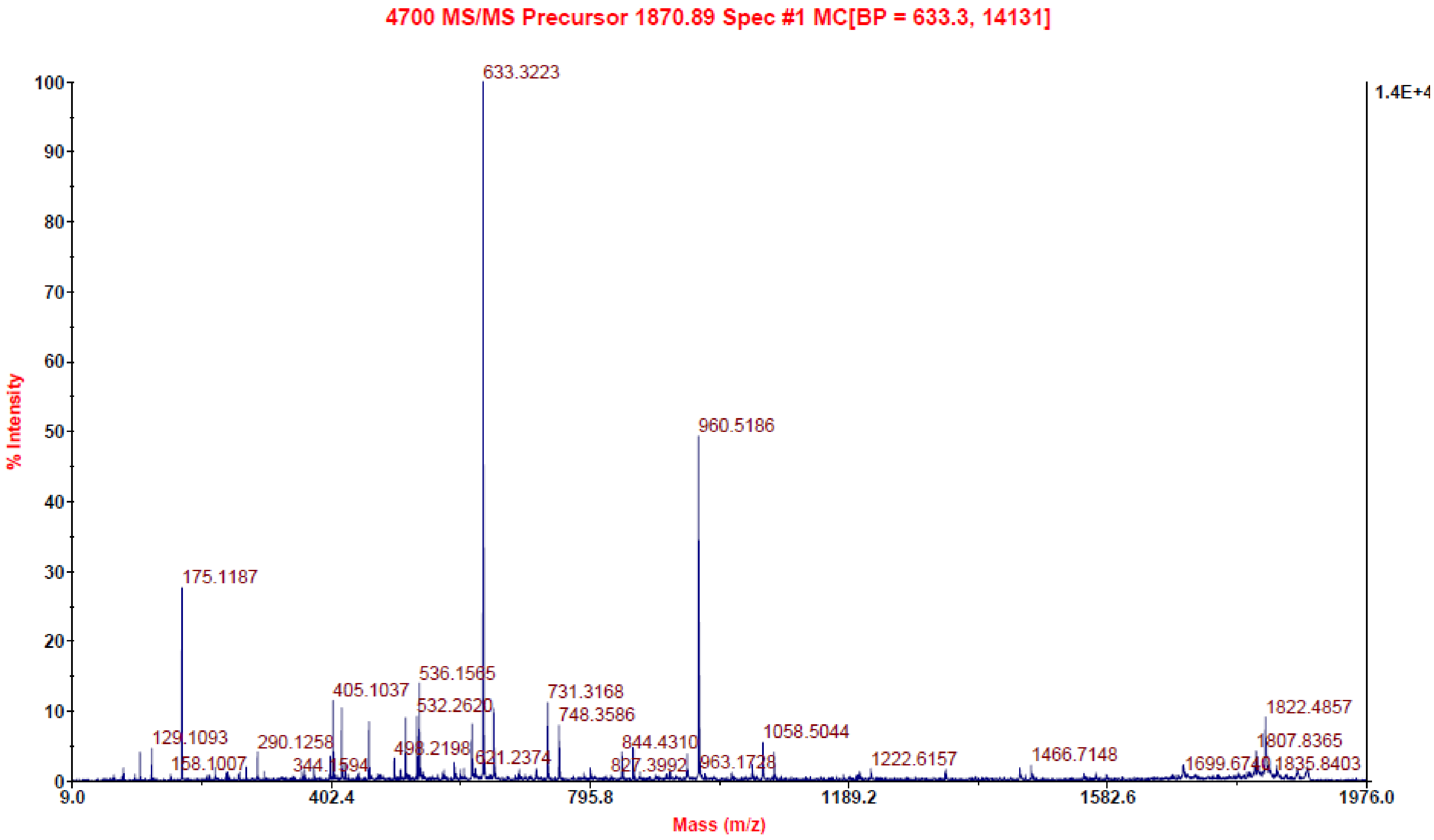

(f)

Figure S1. *Cont.*

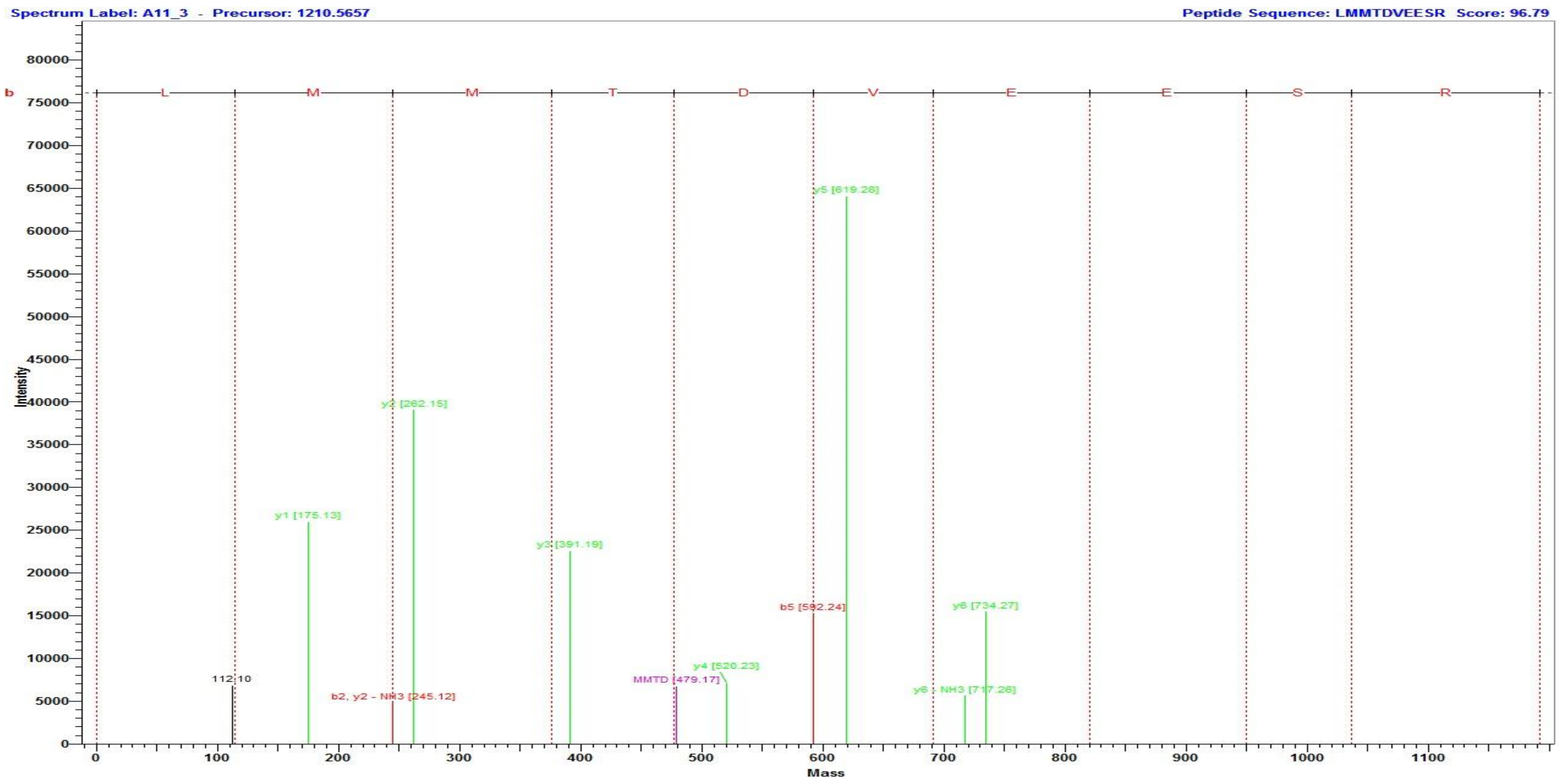

(g)

Figure S1. *Cont*.

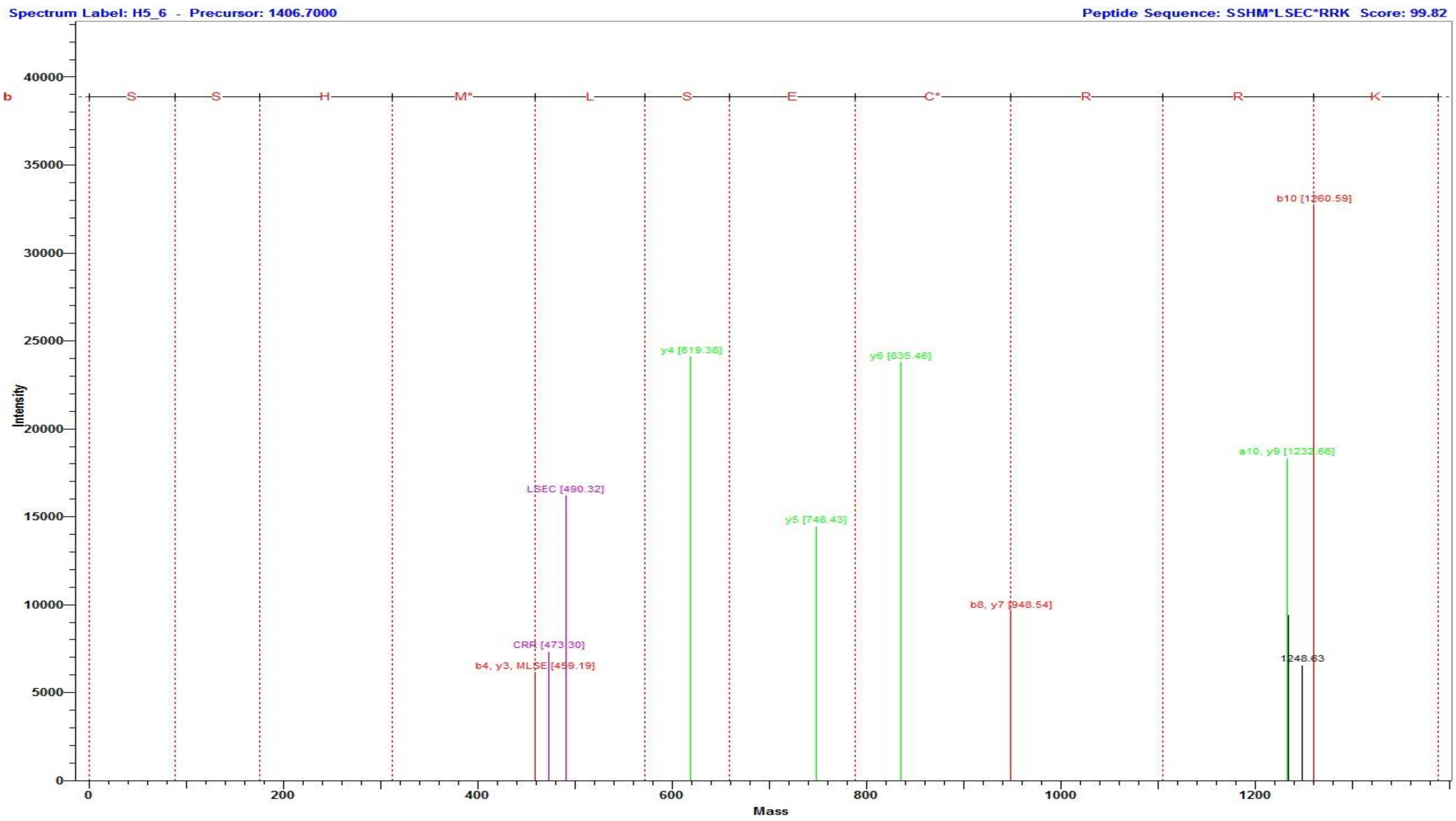

(**h**)

Figure S1. *Cont*.

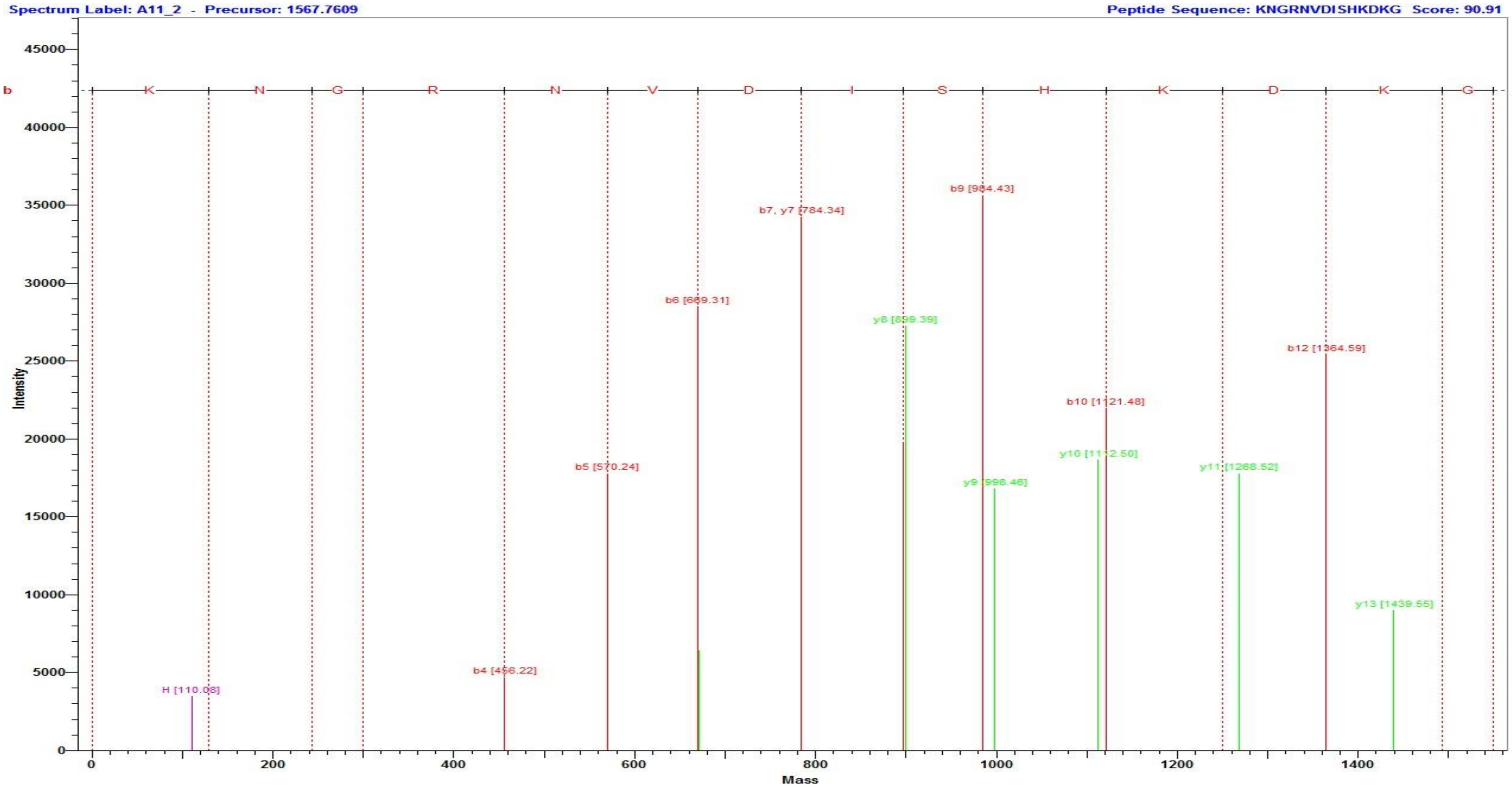

(i)

Figure S1. *Cont.*

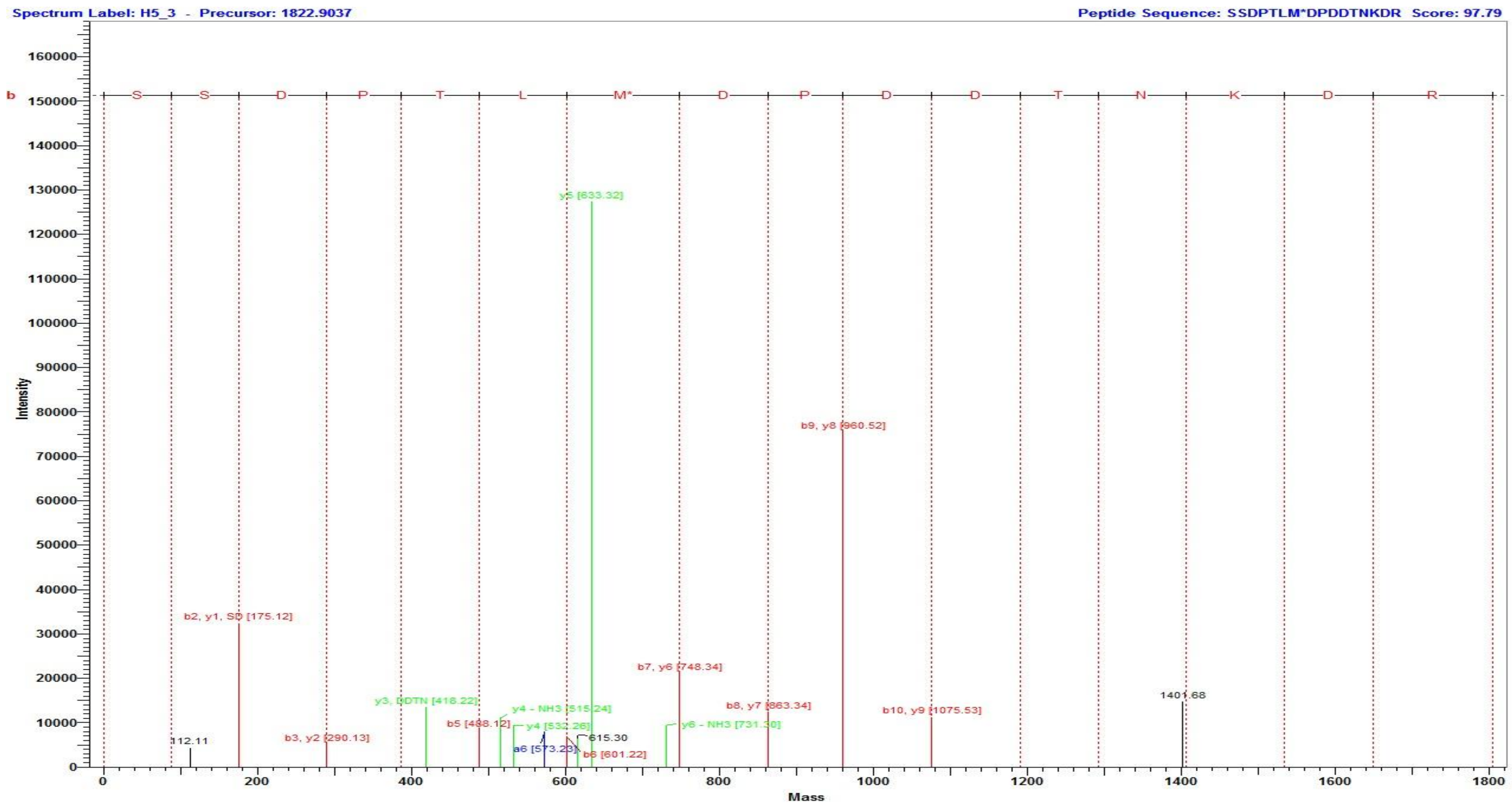

(j)

Figure S1. *Cont.*

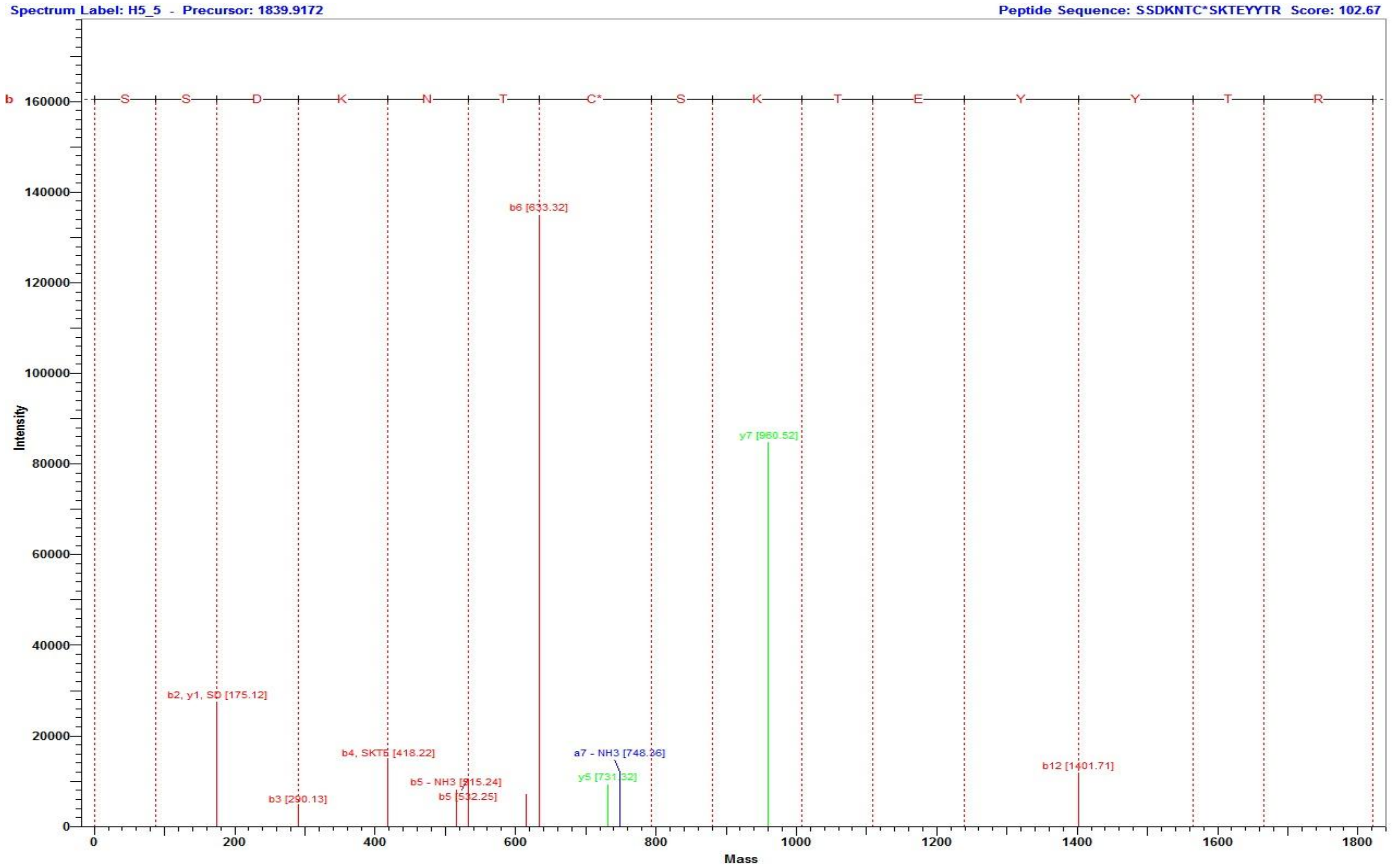

(k)

Figure S1. *Cont.*

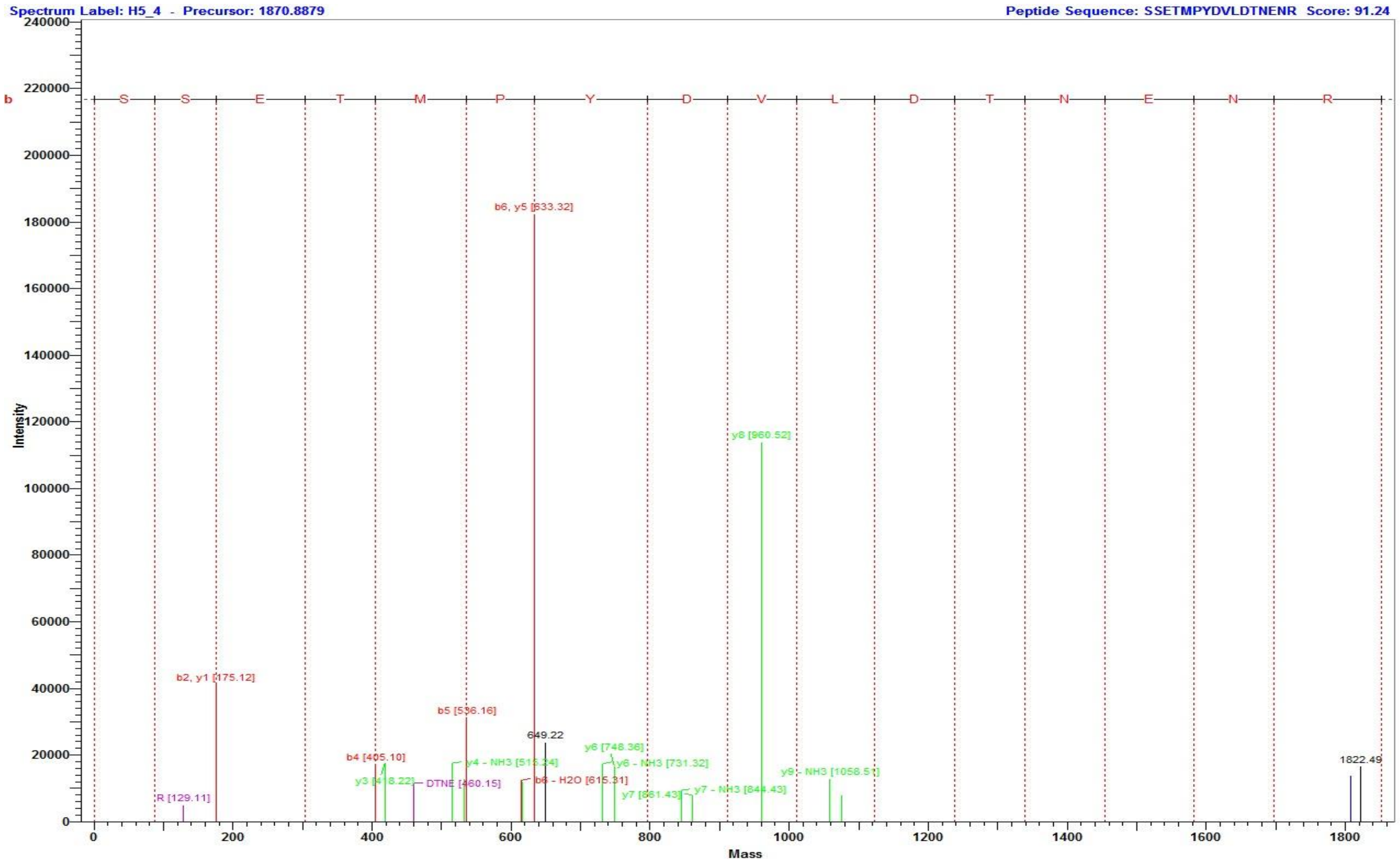

(l)

Figure S1. Peptide mass fingerprinting (PMF) and amino acid sequence of D2-3. (**a**) The MS spectrum of peptide fragmentation 1210.57 Da; (**b**) The MS spectrum of peptide fragmentation 1406.70 Da; (**c**) The MS spectrum of peptide fragmentation 1567.76 Da; (**d**) The MS spectrum of peptide fragmentation 1822.90 Da; (**e**) The MS spectrum of peptide fragmentation 1839.92 Da; (**f**) The MS spectrum of peptide fragmentation 1870.89 Da; (**g**) The amino acid sequence of the peptide 1210.57 Da; (**h**) The amino acid sequence of the peptide 1406.70 Da; (**i**) The amino acid sequence of the peptide 1567.76 Da; (**j**) The amino acid sequence of the peptide 1822.90 Da; (**k**) The amino acid sequence of the peptide 1839.92 Da; (**l**) The amino acid sequence of the peptide 1870.89 Da.

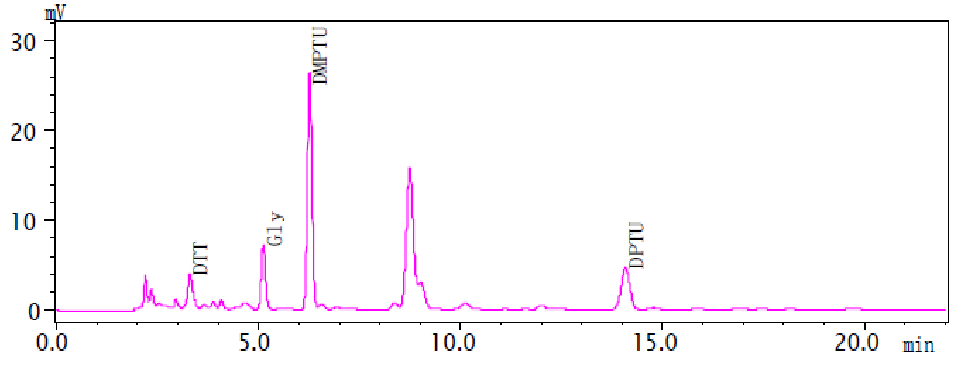

(A)

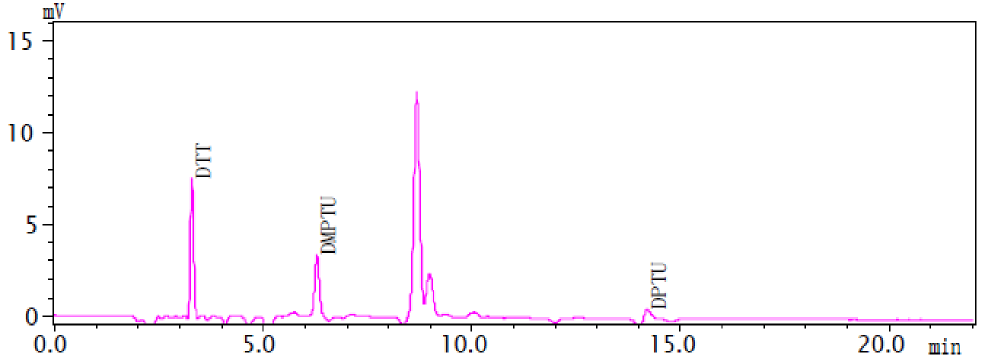

(B)

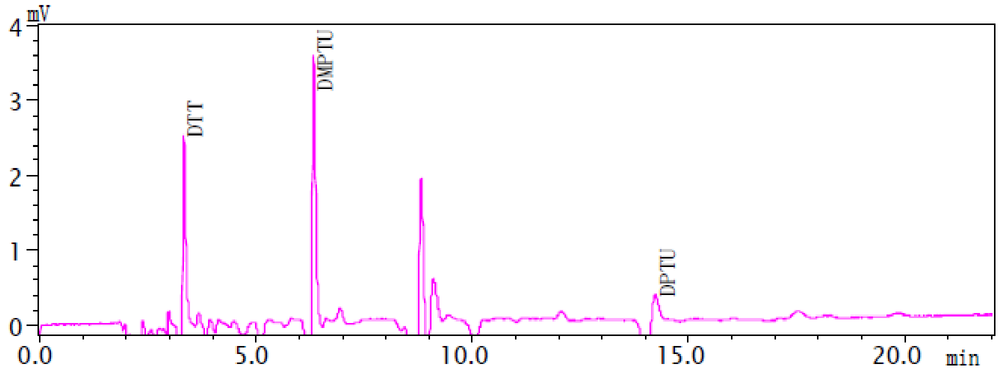

(C)

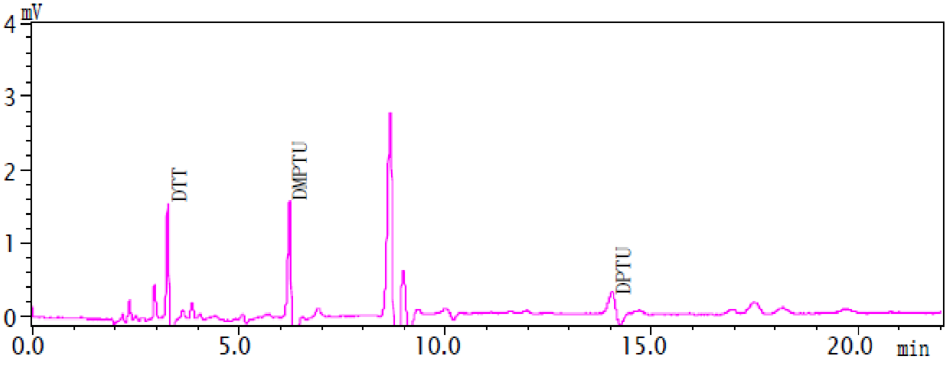

(D)

Figure S2. *Cont.*

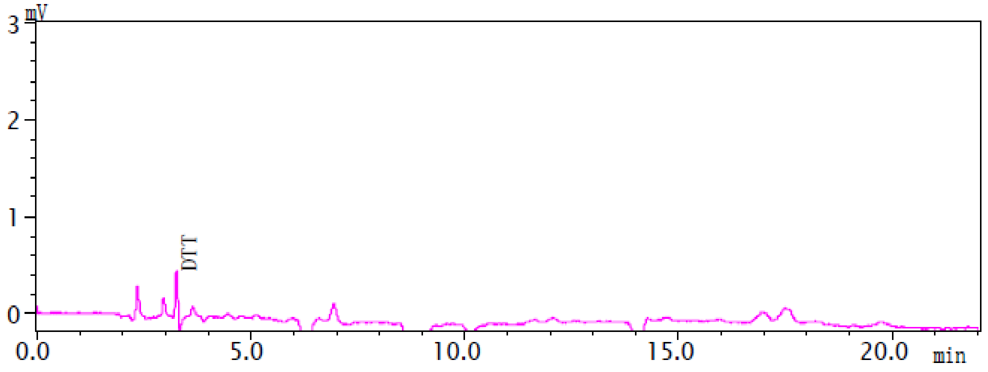

(**E**)

Figure S2. *N*-terminal sequencing by Edman degradation. (**A**) The HPLC chromatogram of Gly. (**B**) The HPLC chromatogram of Pro. (**C**) The HPLC chromatogram of Tyr. (**D**) The HPLC chromatogram of Tyr. (**E**) The HPLC chromatogram of Tyr. (No effective signal peaks were available in the Figure. Five amino acid residues (Gly-Pro-Tyr-Tyr-Tyr) were analyzed, but their amount yield (pmol) and evaluated values were very low. Therefore, we suppose that the *N*-terminus of the purified protein (D2-3) was blocked.)

© 2015 by the authors; licensee MDPI, Basel, Switzerland. This article is an open access article distributed under the terms and conditions of the Creative Commons Attribution license (http://creativecommons.org/licenses/by/4.0/).
